# Factors influencing knowledge on completion of treatment among TB patients under directly observed treatment strategy, in selected health facilities in Embu County, Kenya

**DOI:** 10.11604/pamj.2016.25.234.8761

**Published:** 2016-12-13

**Authors:** Joshua Muriuki Ndwiga, Gideon Kikuvi, Jared Odhiambo Omolo

**Affiliations:** 1 School of Public Health, Jomo Kenyatta University of Agriculture and Technology, P.O.BOX 62000-00200 Nairobi, Kenya; 2 Field Epidemiology and Laboratory Training Program, Ministry of Public Health and Sanitation Kenya P.O. Box 225-00202 Nairobi, Kenya

**Keywords:** Directly observed treatment, treatment completion, Tuberculosis (TB)

## Abstract

**Introduction:**

The World Health Organization (WHO) promotes the Directly Observed Treatment (DOT) strategy as the standard to increase adherence to Tuberculosis (TB) medication. However, cases of retreatment and Multi Drug Resistant continue to be reported in many parts of Kenya. This study sought to determine the factors influencing the completion of tuberculosis medication among TB patients in Embu County, Kenya.

**Methods:**

A descriptive cross-sectional study was conducted on a population of tuberculosis patients under DOT attending selected TB treatment clinics in Embu County, in Kenya. One hundred and forty TB patients interviewed within a period of 3 months. Data were analyzed using SPSS version 17.0 and included Bivariate and Multivariate Analysis. The level of significance was p≤ 0.05.

**Results:**

The male and female participants were 61.4% and 38.6% respectively. The mean age of the respondents was 35±31.34-39.3 years. For the majority (52%) of the participants, the highest level of education was primary education. The unemployed participants formed the highest number of the respondent in the study (73%). The majorities (91.4%0) of the respondents were under the home-based DOT strategy (91.4%, 95% C.I: 85.5-95.5). Bivariate analysis using Chi-square showed that the level of education (p=0.003), patients feeling uncomfortable during supervision (p=0.01), and knowledge regarding the frequency of taking medication (p=0.004) were all significantly associated with knowledge regarding the importance of completion of medication. However, none of these factors was significant after multivariate analysis.

**Conclusion:**

Most participants did not know the importance of completion of medication. TB programs should come up with better ways to educate TB patients on the importance of supervision and treatment completion during the treatment of TB. The education programs should focus on influencing the attitudes of patients and creating awareness about the importance of treatment completion. The TB programs should be designed towards eliminating the factors influencing the completion of TB medication.

## Introduction

The socioeconomic cost of TB is particularly high in developing countries where low quality of life contributes to the spread of TB [1]. In 2013, nearly 2 million people died from TB globally [2]. The Directly Observed Treatment Short-course (DOTS) remains the World Health Organisation standard of care for drug susceptible tuberculosis across the world. However, critics of the DOTS allege that it has no benefits over the alternatives, such as Self-Administered Therapy (SAT) [3]. Under the DOTS program, family members and/or friends observe TB patients taking their medications for the first two months of treatment. The following 4-6 months, patients usually administer their anti-TB drugs in a component called DOT [1]. During the period of unsupervised treatment, some patients default on treatment leading to treatment failure rates, which in turn leads to the spread of TB further and the development of MDR. The Directly Observed Therapy has generated controversies concerning its efficacy in promoting treatment adherence [4]. Conflicting outcomes have been reported about treatment completion under DOT [4].

## Methods

### Study site

The study was conducted in TB treatment health facilities in Embu County, Kenya. The selected facilities included Runyenjes sub-district hospital, Nembure health center, Kianjokoma health center, and Kibugu health center.

### Study design

A descriptive cross-sectional study was used.

### Study population

The study targeted tuberculosis patients over 18 years of age, under treatment in the selected health facilities.

### Sample size determination and sampling

The number of health facilities undertaking TB treatment in Embu County was identified from government records. From these health facilities, random selection of the health facilities included in the study was done. For the selected health facilities, a sampling frame was created using the TB patient records, and stratified random sampling was then used to select 140 patients, under treatment, who were interviewed in the study. The selection and interview of the participants took place within 3 months. The study was conducted between May and July 2011.

### Data collection and analysis

Data were obtained from the respondents using semi-structured questionnaires and entered into to Microsoft Excel for cleaning, coding, and validation. The data were then transferred to the Statistical Package for Social Science (SPSS) for Windows version 17.0 for statistical analysis. Analysis of descriptive statistics, bivariate analysis using Chi-square test, and multivariate logistic regression with a significant level of p≤0.05 was done. Multivariate analysis using Binary Logistic regression was done for those values, significant at the bivariate level.

### Ethical considerations

The study was completed according to the revised Helsinki Declaration [**5**]. Ethical approval was obtained from Kenya Medical Research (KEMRI) Ethical Research Committee. Authorization to conduct the study in the local health facilitated was obtained from the Provincial TB, Leprosy and Lung Disease Coordinator in Eastern Province (Kenya). Authorization was also obtained from the persons in charge of the health facilities that were involved in the study. Moreover, consent was sought from the interviewed patients.

## Results

### Socio-demographic characteristics of the study participants

Most of the study participants were males (61.4%). The mean age of the respondents was 35±31.34-39.3 years. Most (54.5%) of the respondents had attained primary education, followed by secondary (34.1%), college (9.1%) and no formal education (2.3%). Fifty-two (52%) of the participants were unemployed, (20%) were formally employed and (28%) self-employed. The married participants were 49.2%, the single 37.1%, the separated/divorced 8.3%, and the windowed 5.3%. Among the study participants 70.7%, (95% C.I: 62.4-78.1) were HIV negative while 29.3% (95% C.I: 21.9-37.6) were HIV positive. Slightly above half (50.7%) of the study participants travelled 0-1 Km, while 23.6%, 6.4%, 9.3%, and 10% travelled 1-2 Km, 2-3 Km 3-4 Km and >4 Km to access medical services. The majorities of the participants (85.7%) used matatus/buses, 4.3% used bicycles, and 10% walked to the health facilities to access medical services. Majorities (91.4%) of the respondents in this study were under the home-based DOT strategy (91.4%, 95% C.I: 85.5-95.5).

### Factors associated with the likely completion of medication

The factors significantly associated with importance of completion of medication were the highest level of education (post-secondary) (X^2^=13.643 df =3, p= 0.003) and the patients feeling uneasy during supervision (X^2^=6.659 df =1, p= 0.01). Others included the number of times the patients should take medication (Always) (X^2^=10.818, df =2, p= 0.004) and abuse of substance by the respondents (X^2^=13.933 df =5, p= 0.02) (Table 1). The study did not measure completion rate.

**Table 1:** The association between patients’ attitudes toward supervision, with their knowledge on completion of tuberculosis treatment

Variable/Category	Mandatory to complete medication		Chi Square(χ²)	df	P value
	Yes (%)	No (%)			
**Like being Supervised**			2.798	1	0.094
Yes	76 (57.6)	7 (87.5)			
No	56 (42.4)	1 (12.5)			
**Feel uncomfortable during supervision**			6.659	1	0.01
Yes	78 (59.1)	1 (12.5)			
No	54 (40.9)	7 (87.5)			
**Took medication because of supervision**			1.323	1	0.25
Yes	42 (31.8)	1 (12.5)			
No	90 (68.2)	7 (87.5)			
**Supervisor should be present**			4.92	2	0.085
Never/Rarely	14 (10.6)	0 (0)			
Sometimes	70 (53)	2 (25)			
Always	48 (36.4)	6 (75)			
**How often to take medication after prescription**			10.818	2	0.004
Never/Rarely	4 (3)	0 (0)			
Sometimes	30 (22.7)	6 (75)			
Always	98 (74.2)	2 (25)			
**Follow instructions on the tuberculosis treatment**			7.026	2	0.3
Never/Rarely	2 (1.5)	1 (12.5)			
Sometimes	62 (47)	1 (12.5)			
Always	68 (51.5)	6 (12.5)			

Gender was not significantly associated with knowledge regarding the importance of completing tuberculosis treatment (X^2^=2.434 df =1, p= 0.119), Odd Ratio 4.70 (95% C.I: 0.55-104.5). The employment status and the marital status of the participants were not significantly associated with knowledge on the importance of completion of tuberculosis medication (X^2^=4.450 df =2, p= 0.108 and (X^2^=4.772 df =3, p= 0.189) respectively. The distance that the respondents travelled to get to the clinics for tuberculosis treatment and the mode of transport used to get to the health facilities were not significantly associated with patients knowledge on the importance of completion of treatment (X^2^=2.916 df =4, P= 0.572) and 0.42 df=2, P=0.811) respectively. There was no significant association between participants knowledge on whether completion of TB treatment was mandatory with their liking for supervision (X^2^=2.798 df =1, P= 0.094). Taking medication only because a supervisor was present had no significance association with the knowledge on whether completion of medication was mandatory (X^2^=1.323 df =1, P= 0.25). Additionally, the knowledge on whether instructions given by doctors about TB treatment were not significantly associated with their knowledge on the completion of tuberculosis treatment (X^2^=7.026 df =2, P= 0.30). Table 1 has the summary of the results.

There was no significant association between the treatment status (X^2^=1.414 df =2, P= 0.493) and HIV status (X^2^=3.514 df =1, P= 0.061) with whether they knew if completion of tuberculosis was mandatory. The employment status and the marital status of the TB patients were not significantly associated with the knowledge on the importance of completion of mediation (X^2^=4.450 df =2, P= 0.108 and (X^2^=4.772 df =3, P= 0.189) respectively. Table 2 has the summary of the results. The Multivariate Analysis (Binary Logistic regression) indicated that none of the factors had significant association with the TB patients’ knowledge regarding the importance of completion of medication. Table 3 has the summary of the results.

**Table 2:** Socio demographic factors associated with knowledge on completion of treatment

Variable/Category	Mandatory to complete medication	Chi Square(χ²)	df	P value	
	Yes (%)	No (%)			
**The Highest Level of Education**			13.643	3	0.003
No Formal Education	3 (2.3)	1 (12.5)			
Primary	72 (54.5)	0 (0)			
Secondary	45 (34.1)	7 (87.5)			
College	12 (9.1)	0			
**Occupation Status**			4.45	2	0.108
Unemployed	66 (50)	7 (87.5)			
Formally employed	28 (21.2)	0 (0)			
Self-employed	38 (28.8)	1(12.5)			
**Marital Status**			4.772	3	0.189
Married	65 (49.2)	2 (25)			
Single	49 (37.1)	6 (75)			
Separated/Divorced	11 (8.3)	0 (0)			
Widowed	7 (5.3)	0 (0)			

**Table 3:** Multivariate analysis of the factors associated with respondent’s knowledge on the importance of completion of TB medication

Predictor variables	β	S.E. (β)	df	P value	Adjusted	95% C.I. for odds ratio	
					Odds Ratio	Lower	Upper
Age category	1.867	1.064	1	0.079	6.467	0.804	52.044
Level of education	2.404	1.756	1	0.171	11.069	0.354	345.94
Substance abuse	0.574	0.427	1	0.179	1.776	0.768	4.102
Like supervision	-0.58	1.459	1	0.691	0.559	0.032	9.772
Feeling uncomfortable	1.331	1.534	1	0.385	3.785	0.187	76.462
Take medicine because of supervision	1.785	1.64	1	0.276	5.96	0.24	148.312
Think supervisor should be present	0.965	0.768	1	0.209	2.624	0.582	11.83
Follow instructions	0.293	0.639	1	0.646	1.341	0.383	4.69
How often to take medicine	-0.87	0.788	1	0.27	0.419	0.089	1.962

## Discussion

The male respondents enrolled in the study were more compared to the female respondents. The study was contrary to a similar study conducted in Kenya on the use of Technology Assisted DOT. The study enrolled an almost similar number of males (49.7%) to females (50.3%) [6]. However, in a study done in Uganda, males were more compared to the females, 57.1% and 42.9% respectively [7]. Gender was not significantly associated (P=0.152) with the patient’s knowledge on the importance of completion of tuberculosis medication.

In the study, TB was most prevalent in the adults in the 30-39 years age group. These result agree with the WHO 2009 reported that reported that the majority of persons affected by TB are in their most productive years [7–9]. The study participants had attained various levels of education; however, for most participants, primary education was the highest level of education attained followed by secondary education. Similar results were reported in a study done in Uganda, whereby majority of the participants (51%), primary education was the highest level of education followed by secondary school education [7]. The unemployed participants formed the highest category of the respondent in the study (73%), which might be an indication of the poverty level.

The study identified several aspects likely to influence completion of medication among the participants. The results of the study showed that most of the respondents did not like being supervised while taking their medication and more than half of the respondents reported being uncomfortable during supervision. During the in-depth interviews done after a randomized controlled trial in Pakistan, some patients reported that the direct observation of drug taking was unbeneficial and thus they did not comply with the allocated DOT because it inconvenienced them [10]. Moreover, almost 70 percent of the respondents reported that they did not need to be supervised while taking medication. This could be because of the providers’ attitude. In the study done in Pakistan, providers’ views and cynicism were reported as barriers to DOT [10]. The results of the study showed that the patients were knowledgeable about TB treatment. Over 90% of the participants knew the duration of medication, the importance of treatment completion, and articulated that the medication should always be taken.

Several factors were likely to influence patients’ knowledge on the importance of completing tuberculosis medication. In the study, gender did not influence the knowledge regarding the importance of completing tuberculosis treatment. The highest level of education attained by the respondent influenced the knowledge regarding the importance of completing tuberculosis treatment meaning that those that have achieve post-secondary education are more likely to be knowledgeable about the importance of tuberculosis treatment completion. In the study, a significant association was established between the patients feeling uneasy during supervision and the knowledge on whether it was mandatory to complete medication. The result may indicate that the patients who felt uncomfortable during supervision were likely to be unknowledgeable about the need for completing TB medication. Patients who knew the duration of taking TB medication were also knowledgeable about the importance of completing TB medication. In the study, substance abuse and HIV status did not influence the TB medication completion.

The patients interviewed reported three types of the DOT program, including clinic-based (5%), home-based (91.4%), and school-based (3.6%). The majority of those enrolled into the home based program reported that their spouses supervised them as they took their tuberculosis medication. A study done in Tanzania on the patient preference on the type of DOT treatment revealed that 53.1% of the respondents preferred home-based program while 46.9% preferred treatment in the health facilities. In the current study, 52.3% reported being supervised by their spouses. The rest of the respondents had other individuals supervising them In Tanzania, 28% of the TB patients were being supervised by the spouses while 45.6% were being supervised by other family members [11]. The patients receiving first time treatment in this study were 84.8% cases of re-treatment were 13.6%, while the multi-drug treatment cases were 1.5% [11]. In a cross sectional study done in India, 17% of the respondents defaulted on medication during the period of the study [12]. The study aimed to identify the risk factors associated with defaulting, treatment failure and death among TB patients treated under the DOTS program. Santha noted that DOT should be made more convenient for patients because of the high rates of defaulting results in re-treatment cases and MDR [12].

The majorities (94.3%) of the respondents in this study were knowledgeable on the importance of completion of the treatment doses and indicated that completion of the tuberculosis treatment was important. The knowledge on the importance of completion of tuberculosis treatment was the dependent variable in this study. In a study conducted in the Asembo and Gem regions in the Nyanza region, 14 participants were not knowledgeable on the importance of completion of the treatment course in tuberculosis [13]. However, this was a qualitative study with a sample size of 31 respondents. In the current study, only eight of the patients interviewed did not know whether it was mandatory to complete the tuberculosis treatment; perhaps an indicator of the value of education that the patients are given by their health care providers regarding the tuberculosis treatment.

In the study, 50.7% reported that they liked supervision. However, this was not significantly associated with their knowledge on the importance of completion of tuberculosis treatment (P=0.094). Feeling uncomfortable during supervision among the participants was significantly associated with their knowledge on the importance of completion of medication (P=0.01). In this study, 23.2% reported that they took medicine solely because of supervision. In a study done in Ethiopia to establish the attitude of the patients towards supervision under the DOT program, the study found out that the majority (60%) of the participants preferred to work with volunteer community health workers as the supervisors during their treatment under the DOT strategy. Only 12.5% of the respondents preferred to be supervised by the family members [14]. However, the study did not seek to determine how they felt about the supervision.

Another study involving Moroccan tuberculosis patients sought to establish the attitudes and knowledge that they had about the treatment of TB. The study divided the patients into either adherent and non-adherent patients. In both groups, 83.8 % of the patients were knowledgeable about the duration of treatment of TB [15]. In our study, all respondents were knowledgeable about the duration of treatment, (8 months for the re-treatment cases and 6 months for first-time treatment). The Moroccan study inquired about the consequence of lack of treatment completion, 13.8% of the respondents had no idea of the consequences and these were mainly the non-adherent one. Knowledge of the consequence of lack of completion of treatment was significantly associated (P=0.01) with adherence to tuberculosis treatment [15]. The findings of the study indicated that 94.3% of the respondents were knowledgeable about the completion of the treatment.

## Conclusion

The majority of the respondents in the study were knowledgeable on the importance of completion of tuberculosis treatment. The factors that influenced completion of tuberculosis medication in the study included the level of education, uneasiness during supervision, knowledge of the required frequency in taking medication, and knowledge on the importance of following instructions. **Recommendations:** enhancing the quality of education given to TB patients once they are started on medication, offering counselling services on the importance of completing treatment on a regular basis, and the reasons it is a mandatory to complete treatment can help reduce retreatment cases.

### What is known about this topic

- Level of income influences completion of tuberculosis treatment among patients;
- Knowledge about tuberculosis influences medication completion rate among patient.

### What this study adds

- The current TB education programs are inadequate to impart the necessary knowledge on TB patient regarding the importance of treatment completion;
- Feeling uneasy/uncomfortable during supervision while taking medication influences treatment completion.
